# Beta Oscillations Distinguish Between Two Forms of Mental Imagery While Gamma and Theta Activity Reflects Auditory Attention

**DOI:** 10.3389/fnhum.2018.00389

**Published:** 2018-09-25

**Authors:** Mario Villena-González, Ismael Palacios-García, Eugenio Rodríguez, Vladimir López

**Affiliations:** ^1^Laboratorio de Psicología Experimental, Escuela de Psicología, Pontificia Universidad Católica de Chile, Santiago, Chile; ^2^Laboratorio de Neurociencia Cognitiva y Social, Facultad de Psicología, Universidad Diego Portales, Santiago, Chile; ^3^Laboratorio de Neurodinámica, Escuela de Psicología, Pontificia Universidad Católica de Chile, Santiago, Chile

**Keywords:** beta band activity, auditory attention, mental imagery, self-generated thoughts, theta band activity, gamma band activity

## Abstract

Visual sensory processing of external events decreases when attention is internally oriented toward self-generated thoughts and also differences in attenuation have been shown depending on the thought’s modality (visual or auditory thought). The present study aims to assess whether such modulations occurs also in auditory modality. In order to investigate auditory sensory modulations, we compared a passive listening condition with two conditions in which attention was internally oriented as a part of a task; a visual imagery condition and an inner speech condition. EEG signal was recorded from 20 participants while they were exposed to auditory probes during these three conditions. ERP results showed no differences in N1 auditory response comparing the three conditions reflecting maintenance of evoked electrophysiological reactivity for auditory modality. Nonetheless, time-frequency analyses showed that gamma and theta power in frontal regions was higher for passive listening than for internal attentional conditions. Specifically, the reduced amplitude in early gamma and theta band during both inward attention conditions may reflect reduced conscious attention of the current auditory stimulation. Finally, different pattern of beta band activity was observed only during visual imagery which can reflect cross-modal integration between visual and auditory modalities and it can distinguish this form of mental imagery from the inner speech. Taken together, these results showed that attentional suppression mechanisms in auditory modality are different from visual modality during mental imagery processes. Our results about oscillatory activity also confirm the important role of gamma oscillations in auditory processing and the differential neural dynamics underlying the visual and auditory/verbal imagery.

## Introduction

When attention is internally oriented toward self-generated thoughts (SGT), the sensory/cognitive processing of external events decreases, which is known as perceptual decoupling (Smallwood and Schooler, 2006). This decoupling can be observed as impairments in performance during demanding tasks as well as reductions in amplitude of the ERP components related with sensory response (Smallwood and Schooler, 2015).

Studies focused on visual response reduction during SGT have shown consistent results regarding this sensory attenuation. For instance, they have shown similar ERP amplitude reductions in P1 and P300 components, regardless of the experimental paradigm (Smallwood et al., 2008; Baird et al., 2014; Barron et al., 2011; Kam et al., 2011).

However, for auditory processing during SGT the evidences are rather inconclusive. On the one hand, studies have shown reductions in auditory N100 during mind wandering contrasted with on-task conditions (Kam et al., 2011; Kam and Handy, 2013). Nonetheless, Kam et al. (2013) also found maintenance of auditory reactivity toward deviant stimuli, as an adaptive mechanism to respond to environment even during a mind wandering episode (also see: Kam and Handy, 2013). On the other hand, Braboszcz and Delorme (2011) showed attenuation of the auditory ERP during mind wandering in a different time window; over the 200 ms post stimulus.

This non-conclusive evidence leave the open question of whether the auditory modality uses different attentional suppression mechanisms compared to visual modality and whether there is maintenance of auditory sensitivity when attention is internally oriented. Furthermore, if there is actually an attentional suppression process, it is not clear yet if specific mental states can modulate auditory attention during SGT. For instance, visual processing has been shown to be differentially affected depending on the thought’s modality, i.e., visual or verbal/auditory thoughts, and this was showed for ERP and alpha oscillations (Villena-González et al., 2016). Assessing whether thought’s modality (visual or auditory) affects in the same way the sensory response in the visual and auditory cortices would provide important information about supra-modal or modality specifics mechanisms of attentional suppression and also to better understand to what extent SGT might affect our daily attentional performance.

Given that brain oscillations can provide an understanding about levels of processing of the brain function different from ERPs (Cohen, 2014a), previous research has also investigated the functional association between SGT and brain oscillations, showing changes at different frequency bands (alpha, beta, theta) when comparing SGT with external tasks (Cooper et al., 2003; Engel and Fries, 2010; Braboszcz and Delorme, 2011). On the other hand, gamma band activity has been classically shown to index auditory stimuli processing (Tiitinen et al., 1993; Cervenka et al., 2011).

The present work aims to assess whether auditory response is attenuated when attention is oriented toward internal thoughts, but also if there is any differential perceptual processing associated to the modality of the thought (visual or auditory/verbal). For this purpose, we used the auditory N1 component of ERPs and time-frequency measures in order to evaluate auditory sensory processing when participants performed three conditions; one in which participants passively listened auditory stimulation, one in which they performed a visual imagery task and the last one in which they were asked to perform an inner speech task. We hypothesize that during passive listening condition there should be a greater processing of auditory stimuli than during internal thoughts, which would be reflected as a larger auditory N1 and more spectral power in the gamma band during passive listening than in the other conditions. When both imagery conditions are compared (inner speech vs. visual imagery), we hypothesize that there should be a greater processing of auditory stimuli during visual imagery than during inner speech, given that during inner speech the auditory processing resources are being used by the internal task. This hypothesis is based on the theoretical framework proposed by Villena-González et al. (2016) in which there would be a processing resource competition if the modality of thought is the same that incoming stimulation. This would be reflected as a larger auditory N1 and more spectral power in the gamma band during visual imagery than during inner speech condition.

## Materials and Methods

### Participants

Twenty volunteers (9 women) were recruited for the study (mean age = 23, range = 18–30). All participants had normal or corrected-to-normal vision, and reported no color-vision deficiency. Participants had no history of drug abuse, neurological or psychiatric conditions. The protocol was approved by the Ethics Committee of Pontificia Universidad Católica de Chile. All subjects gave written informed consent in accordance with the Declaration of Helsinki. All experiments were performed at the Neurodynamic Laboratory of the School of Psychology of this University.

### Stimuli and Procedure

**FIGURE 1:**
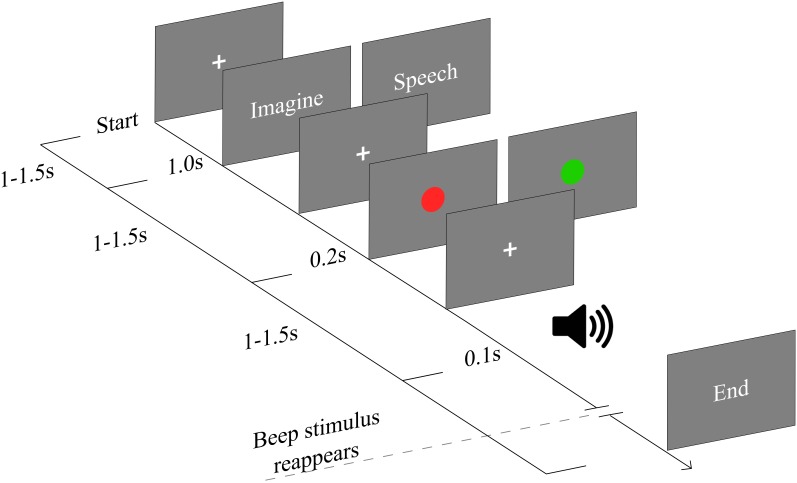
Schematic of experimental paradigm. Each trial started by pressing a button triggering a fixation cross presented between 1 and 1.5 s. When the word “imagine” appears followed by the green circle, participants were instructed to think about any mental image, avoiding auditory elements. If the word “speech” was followed by a green circle participants were instructed to think using only their inner speech. Finally, if any of the two words were followed by a red circle, participants were instructed to passively hearing the auditory stimuli. Color cues were counterbalanced between blocks. Auditory probes are presented in each condition (1200 Hz, 75 dB SPL) with duration of 100 ms. The inter stimulus interval was randomly jittered between 1000 and 1500 ms.

All stimuli were presented on a computer screen with gray background situated 57 cm away from the participant. Psychopy software (Peirce, 2007) was used to design the experiment and display the stimuli. The task is a modified version of the task used in Villena-González et al. (2016). The task was composed of 2 blocks; each block had 30 auto-administrated trials. Before each block participants were asked to fix their eyes on a fixation cross in the middle of the screen. Participants started each trial by pressing a button. After this, either the word “imagine” or “speech” came up in the middle of the screen (**Figure 1**). Afterwards, a colored circle (2 cm radius) appeared indicating whether the attention should have been allocated in the external stimuli (red circle) or in their thoughts (green circle). When the word “imagine” appeared followed by the green circle, participants were instructed to think about anything they wanted, given that it had a strictly visual quality, avoiding auditory or phonological elements of any form. This condition will be named throughout the text as: “visual imagery.” If the word “speech” was followed by a green circle participants were instructed to think using their inner speech without using any sort of mental image. This condition will be referred to as: “inner speech.” Finally, if any of the two words were followed by a red circle, participants were instructed to covertly attend the appearing tone beeps. This condition will be referred to as “passive listening.” After the circle appearance, during the passive listening or imagery period, 15 auditory probes (beep tone; 1200 Hz, 75 dB SPL) were presented through headphones with duration of 100 ms. The inter stimulus interval was randomly jittered between 1000 and 1500 ms. Color circles were counterbalanced, i.e., during the second block the red circle cued the thought related tasks and the green circle cued the auditory related task.

### EEG Recording

EEG data was obtained using 64 electrodes (Biosemi^®^ ActiveTwo) arranged according to the international 10/20 extended system. Horizontal and vertical eye movements were monitored using four external electrodes. Horizontal EOG was recorded bipolarly from the outer canthi of both eyes and vertical EOG was recorded from above and below of the participant’s right eye. Two additional external electrodes were placed on the right and left mastoid to be used for later re-referencing.

### EEG Data Pre-processing

Data pre-processing was performed using Matlab 7.8.0 (The Mathworks, Inc.) with EEGLAB v7.1.7.18b toolbox (Delorme and Makeig, 2004). The signal was down-sampled off-line at 1024 Hz. Because of hardware setup constraints, all electrodes were referenced to CMS and DRL during acquisition, but off-line re-referenced to averaged mastoids. Horizontal and Vertical EOG were calculated by means of the difference between left–right electrodes and above–below electrodes, respectively. For ERP analysis, a 2nd order infinite impulse response (IIR) Butterworth filter was used for band-pass filtering continuous EEG data, with a half amplitude cut-off frequency of 0.05 Hz and 30 Hz. For frequency analyses we used a band-pass filtering on epoched EEG data with a half amplitude cut-off frequency of 0.5 and 80 Hz using Fieldtrip software (Oostenveld et al., 2011). Artifact detection was performed on segmented data (see below epoch segmentation details) by manual inspection blind to condition. All epochs with artifacts were rejected.

### ERP Calculation

The EEG signal was segmented into 20 trials per conditions; each trial window was captured from the appearance of the colored circle up to the trial’s end. Further segmentation in epochs was applied for each condition, selecting the 200 ms preceding each auditory probe appearance up to 500 ms after that. All of the epochs corresponding to the first appearing auditory probe were discarded given that participants were less likely to have already engaged in the thought construction process. After epochs rejections due to artifacts, the total amount of epochs per condition was in average 168.4 (*SD*: 27.69) for Passive listening, 163.95 (*SD*: 36.42) for Inner speech and 168.55 (*SD*: 31.62) for Visual imagery.

Epochs were averaged for each participant and condition. The middle-latency auditory response was defined as the local peak (Luck, 2005) occurring in the 70–90 ms time windows following the auditory probe (P1). Two time windows were calculated for N1 negative deflection; 90–110 ms and 130–160 ms. The Fz, FCz, Cz, CPz, and Pz electrodes were used for this computation given that midline fronto-central electrodes are widely known to be the best location to better capture the auditory N1 effect (Kam et al., 2011).

### ERP Statistics

Local peak amplitude of P1, N1 (90–110 ms), N1 (130–160 ms) evoked by auditory probes were compared with repeated-measures ANOVA using Electrode (five levels: Fz, FCz, Cz, CPz, and Pz) and Condition (three levels: visual imagery, inner speech, passive listening) as factors. All ANOVA analyses were carried out using STATISTICA 7.0 software (StatSoft, Inc.).

### Time-Frequency Calculation and Baseline Normalization

Epochs time-locked to auditory stimulus were computed between −500 and 1000 ms. Afterwards we applied the short-time fast Fourier transform (FFT) method for extracting time-frequency power to each of these epochs. FFT was applied to sequential and overlapping segments of 250 ms of signal tapered by a Hanning taper in order to minimize the possibility of edge artifacts. The amount of overlap between successive time segments was 25 ms. Thus, spectral power was computed for every frequency bin (between 1 and 80 Hz). This procedure was performed for each epoch, electrode and participant, and then it was averaged across epochs. In order to eliminate evoked power, the average across epochs in the time domain was subtracted from each epoch in the matrix before the time-frequency transformation, in order to obtain the induced power time-frequency chart. Finally, the resulting signal was normalized by converting it to a *Z*-score relative to the baseline time windows. Using this *Z*-normalization, power data are scaled to standard deviation units relative to the power data during the baseline period. All these analyzes were computed using Fieldtrip software (Oostenveld et al., 2011).

### Electrodes Selection for Time-Frequency Statistics

Electrodes were chosen using the data-driven methodology described by Keil et al. (2014) in order to follow an unbiased approach. According to this procedure, we averaged across all conditions, time, frequency and participants in order to reveal the topographical distribution of power in the scalp. Based on power values of the electrodes, a threshold was set in order to keep with the 10% of electrodes with the higher values of power. Two clusters were identified; a frontal and a parietal one. Specifically, we took a cluster of six electrodes over the frontal region (Fpz, Fp1, Fp2, AFz, AF3, and AF4) and another cluster of three electrodes in the parietal region (CPz, P1, and P2) (**Supplementary Figure S3**). These two clusters were used to perform the time-frequency statistical analysis.

### Permutation Test and Multiple Comparison Correction

Time-frequency charts were averaged across epochs resulting in a grand average time–frequency chart for each experimental condition, participant and electrode cluster. For each electrode cluster (frontal and parietal), differences of spectral power were assessed using a non-parametric randomization test, including a correction for multiple comparisons (Nichols and Holmes, 2002; Maris and Oostenveld, 2007). The randomization (or permutation) test is a procedure in which the time-frequency charts belonging to different conditions are shuffled to compute a random distribution. This is then used to evaluate the statistical significance of the results by testing that the experimental differences between conditions exceed the random distribution (null hypothesis distribution). Following this rationale, the first step of the permutation test was to shuffle time-frequency charts across conditions and randomly choose two groups from the complete sample and calculate a *t*-statistic for each time-frequency bin. This step was repeated 1000 times. To correct for multiple comparisons, the highest *t*-value of each permutation was included in the permutation distribution (Bosman et al., 2012; Cohen, 2014b). Finally, from this distribution, the 5th percentile threshold value was used as a threshold to compare the permutation distribution with the original *t*-values. All values above this threshold were considered statistically significant with a *p* < 0.05.

## Results

The main aim of the present study was to investigate whether the auditory response is attenuated when attention is inwardly oriented as it has been reported for visual modality. Secondly, we assessed whether differences in mental representation can influence the potential attenuation of the sensory response. For these two purposes, we analyzed ERPs components and time-frequency power spectrum, while participants passively listened auditory stimuli, performed visual imagery or while they generated an inner speech.

### Early Components of Auditory ERP

**FIGURE 2:**
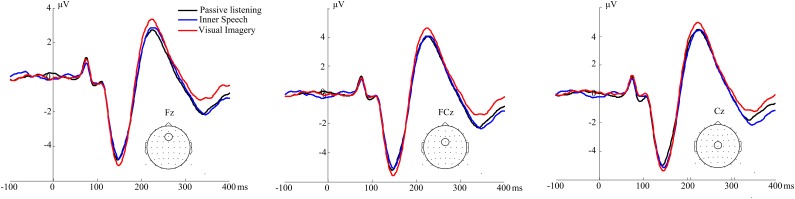
ERPs to auditory probe. ERP waveforms for three different midline electrodes; Fz, FCz, and Cz. There are not differences between conditions for any of the early sensory components of ERP.

We analyzed the ERP evoked by the auditory probe. The amplitude for the different voltage deflections were calculated in five mid-line electrodes (Fz, FCz, Cz, CPz, and Pz) for each participant and condition. Repeated measure ANOVA showed no differences between conditions for any of the analyzed ERP components. P1 [*F*(2,38) = 1,88, *p* = 0.166], N1 (90–110 ms) [*F*(2,38) = 0.216, *p* = 0.806], N1 (130–160 ms) [*F*(2,38) = 0.62562, *p* = 0.540]. ERP waveform is shown in **Figure 2** and **Supplementary Figure S1**. Scalp topography of the N1 and P2 components are shown in **Supplementary Figure S2**.

### Time-Frequency Results

In order to investigate whether the different attentional states affects oscillatory brain dynamics, we calculated the time-frequency charts corresponding to the spectral power induced by auditory stimulus in different frequency bands. This analysis was performed in two different clusters of electrodes from frontal and parietal regions. All differences reported in the following section are significant after multiple comparison correction described in Section “Permutation Test and Multiple Comparison Correction” for non-parametric permutation test (*p* < 0.05).

#### Time-Frequency Results in the Frontal Area

**FIGURE 3:**
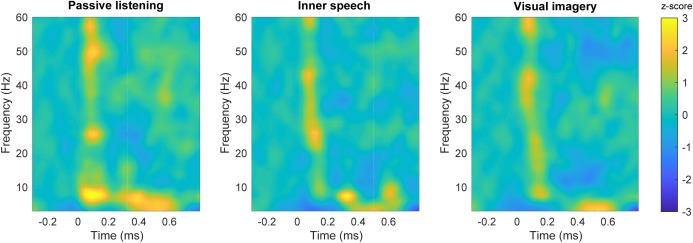
Time-frequency plots in the frontal area. Time-frequency plot of spectral power for each condition; passive listening **(left)**, inner speech **(middle)** and visual imagery **(right)**.

**FIGURE 4:**
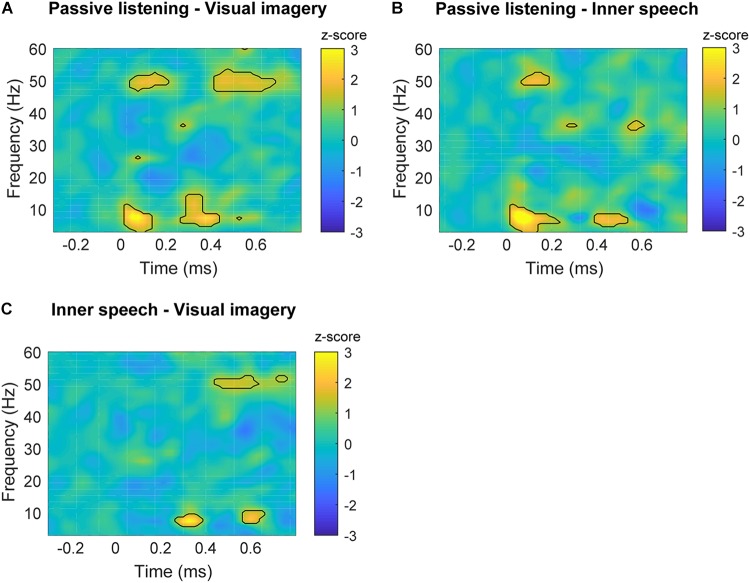
Time-frequency plots of significant differences between conditions in frontal areas. **(A)** Time-frequency power subtraction between passive listening and visual imagery. **(B)** Time-frequency power subtraction between passive listening and inner speech. **(C)** Time-frequency power subtraction between inner speech and visual imagery. The areas delimited by lines show the frequency and time range in which significant differences are found after multiple comparison correction described in Section “Permutation Test and Multiple Comparison Correction” for non-parametric permutation test (*p* < 0.05).

Time-frequency plots are shown for each experimental condition in **Figure 3**. Differences in spectral power can be observed for gamma and theta frequency bands when passive listening was compared with internal attentional conditions (**Figures 4A,B**). Specifically, a narrow band (48–53 Hz) within gamma range showed a higher spectral power during the passive listening condition contrasted with the other conditions, between 70 and 200 ms. Another effect in gamma band (46–54 Hz) can be observed later in time between 420 and 670 ms, showing higher gamma power during passive listening than in visual imagery condition (**Figure 4A**). This late effect can also be observed when inner speech is compared with visual imagery (**Figure 4C**). In this case gamma band power (48–52 Hz) is higher in inner speech than in visual imagery during the 450–620 ms time window.

Differences in low frequencies can also be observed, with the passive listening condition showing higher power than inner speech between 3 and 10 Hz (25–200 ms) and later between 4 and 8 Hz (400–550 ms). Besides, passive listening condition also showed higher power than visual imagery in a very similar frequency band and time window, early between 3 and 10 Hz (25–150 ms) and later between 5 and 14 Hz (300–400 ms). These differences can also be observed in **Figures 4A,B**.

Finally, inner speech condition showed a higher power than visual imagery between 6 and 10 Hz during 270–400 ms and 570–670 ms (**Figure 4C**). Time-frequency charts of non-significant comparisons are in **Supplementary Figure S4**.

#### Time-Frequency Results in the Parietal Area

**FIGURE 5:**
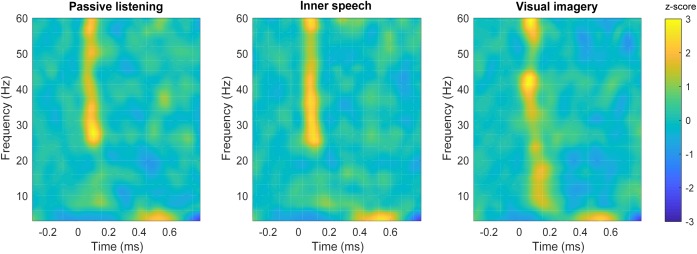
Time-frequency plots in the parietal area. Time-frequency plot of spectral power for each condition; passive listening **(left)**, inner speech **(middle)**, and visual imagery **(right)**.

**FIGURE 6:**
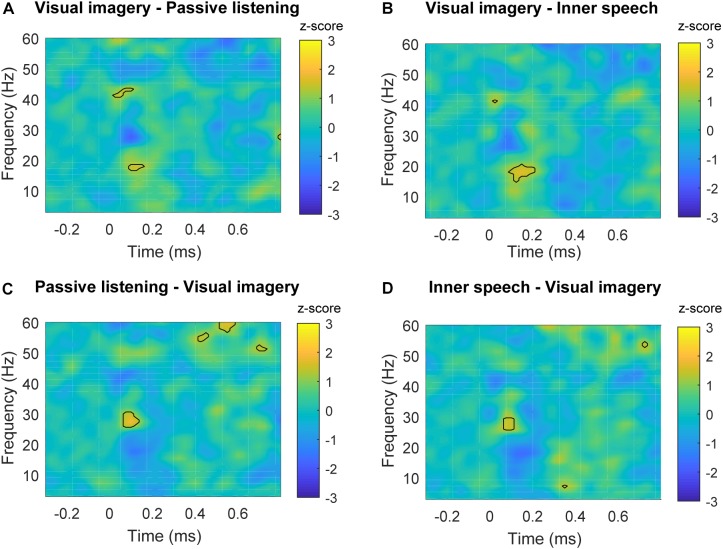
Time-frequency plots of significant differences between conditions in parietal areas. **(A)** Time-frequency power subtraction between visual imagery and passive listening. **(B)** Time-frequency power subtraction between visual imagery and inner speech. **(C)** Time-frequency power subtraction between passive listening and inner speech. **(D)** Time-frequency power subtraction between inner speech and visual imagery. The areas delimited by lines show the frequency and time range in which significant differences are found after multiple comparison correction described in Section “Permutation Test and Multiple Comparison Correction” for non-parametric permutation test (*p* < 0.05).

Time-frequency plots of parietal region are shown for each experimental condition in **Figure 5**. Differences in spectral power can be observed for beta frequency bands when visual imagery was compared with the other conditions. Specifically, visual imagery showed higher power than inner speech between 15 and 20 Hz (100–200 ms) and also higher than passive listening between 16and 19 Hz (100–150 ms) (**Figures 6A,B**). Interestingly, visual imagery showed lower power in the same temporal range but in higher range of beta band (**Figures 6C,D**). Specifically, visual imagery showed lower power than passive listening between 26 and 30 Hz (70–150 ms) and also lower power than inner speech between 26 and 29 Hz (70–120 ms). As it can be observed in **Figure 5** and **Supplementary Figure S5**, there is an increase in beta band activity in all the conditions after the auditory stimulus, but the increase is restricted to high beta during passive listening and inner speech while during visual imagery the increase is in the entire range of beta broadband. Finally, visual imagery showed higher power than passive listening between 41 and 43 Hz (25–100 ms) and passive listening showed higher power than visual imagery between 53–56 Hz (420–470 ms), 56–62 Hz (520–600 ms), and 51–53 Hz (680–720 ms).

## Discussion

The present study sought to assess whether the auditory response is attenuated when attention is inwardly oriented toward mental imagery. Secondly, we also investigated if differences associated to the modality of the thought (visual or auditory/verbal) affects the brain auditory processing. ERPs and time-frequency power were analyzed while participants passively listened auditory stimuli, performed visual imagery or while they generated an inner speech.

The results of the present work showed no differences for early components of the auditory ERP between conditions. However, time-frequency analyses showed higher frontal gamma and theta power during passive listening than in the internal attentional conditions. We also found a different pattern of beta band activity for the visual imagery condition contrasted with the other two conditions. In the following we will discuss in detail these results.

### ERPs Showed Maintenance of Auditory Evoked Response During Mental Imagery

Auditory ERPs were measured in order to assess sensory response to external auditory stimuli. In the present study we did not find differences between passive listening and inward attention conditions, neither when both inward conditions were compared. This result showed that when participants are instructed to orienting their attention to either visual or auditory thoughts, there is a maintenance of auditory sensory response, which suggests this modality operates differently from visual modality regarding attentional suppression mechanisms and controvert some previous findings about this issue.

The sensory attenuation during self-generated thoughts has been reported and widely replicated for visual modality (Smallwood et al., 2008; Barron et al., 2011; Kam et al., 2011; Baird et al., 2014). However, the sensory attenuation for auditory modality still remains unclear since the sensory modulations reported in previous studies have been inconclusive. For instance, studies have shown reductions in auditory N100 during mind wandering contrasted with on-task conditions (Kam et al., 2011, 2013). Interestingly, the same authors also found the auditory modality maintain the response toward deviant stimulus. They claimed this maintenance is an adaptive mechanism which is useful to respond to potentially dangerous environment stimuli even when participants are engaged in mind wandering (Kam and Handy, 2013). This latter claiming suggests auditory modality would have a flexible attentional suppression mechanism, varying depending on the kind of stimuli. On the other hand, Braboszcz and Delorme (2011) showed amplitude attenuation of auditory ERP over 200 ms post stimulus during mind wandering contrasted with on task, but differences in early N1 were not found. These differences can be attributed to differences in the task and the setup of each experiment, which it was proposed in a posterior review about the issue (Kam and Handy, 2013). Following this rationale, the absence of differences in the present study can be due the auditory system is deploying a flexible attentional suppression mechanism depending on context variation (e.g., type of task) but it could be due also because of factors such as psychological variables, for instance, the deliberateness of thought. In the mentioned previous works, SGT was measured as a spontaneous mind wandering but in the present study the attention was deliberately oriented toward thoughts as a part of a task. Deliberateness/spontaneity distinction is starting to be strongly considered in the field of self-generated thoughts (Seli et al., 2016a,b) since this is an important psychological variable that could influence many aspect of brain activity such as functional connectivity between attentional fronto-parietal network with default mode network (Golchert et al., 2017). Another possibility is that auditory system is differentially affected depending on the type of stimulation. For example, Bekinschtein et al. (2009) designed an auditory paradigm that assessed brain responses to violations of temporal regularities that were either local in time or global across several seconds. Local violations led to an early response in auditory cortex, independent of the focus of attention or the presence of a concurrent visual task, whereas global violations led to a late and spatially distributed response that was only present when subjects were attentive and aware of the violations (Bekinschtein et al., 2009). In this sense, auditory system can be prone to be attentional suppressed during SGT only for stimulation with some degree of complexity and temporality rules/patterns. This might be due an adaptation mechanism of human auditory system in order to allowing language processing (Aboitiz, 2012), which require the processing of temporal complex and global constructions rather than merely single tones without any pattern (as in the case of the present study). Taking all this evidence into account, the auditory modality maintain the reactivity toward external stimuli measured with ERPs when attention is deliberately oriented to internal thought as a part of a task, which can be explained by the intentionality of the process or the nature of stimulation. From the ERP results in the present study it is not possible to quite understand the mechanisms underlying attenuation in auditory system and further ERP research is needed to specify the conditions under which these differences can be observed.

### Early Gamma and Theta Oscillations in Frontal Areas Reflect Differences Between External vs. Internal Attention

Given that the induced brain oscillations reflect different levels of processing of the brain function compared with evoked time-domain techniques (Cohen, 2014a), we assessed whether auditory processing elicits changes in brain oscillations depending on whether the attention was externally or internally oriented. Thus, we analyzed the time-frequency spectral power in different frequency bands.

Our results showed higher frontal gamma and theta power during passive listening than in the internal attention conditions. Specifically, early frontal gamma band activity showed a higher power during passive listening when it was contrasted with the other two conditions (**Figures 4A,B**). This result is in line with previous studies showing that selective attention and top-down attentional processing in auditory modality can modulate the early gamma band activity (Tiitinen et al., 1993; Debener et al., 2003). Tiitinen et al. (1993) showed that gamma power is higher between 25 and 100 ms post-stimulus when attention is focused on the auditory stimuli, contrasted with when participants were unattended or engaged in a competing task (reading a book). Gamma band power has been also observed to increase in conditions of conscious perception contrasted with not perceived stimulus (Rodriguez et al., 1999; Castelhano et al., 2013). However, in the present study we were not able to confirm this point (for instance, we do not have behavioral measures of perceived beeps vs. unperceived beeps). Despite of this, our results suggest that the enhanced gamma activity reflects conscious attention to the auditory stimuli during passive listening which is not present in the other two conditions. Importantly, it has been shown that, besides auditory N100, gamma activity can provide further and complementary information about auditory processing, supporting our pattern of results (Cervenka et al., 2011). Regarding the difference in late gamma oscillation between visual imagery and the other two conditions (**Figures 4A,C**), it can be observed that late gamma slightly increase around 400 ms in a very similar way during passive listening and inner speech but it becomes slightly negative during visual imagery which causes the significant differences (**Figure 3**). This pattern might be interpreted as a further attentional suppression mechanism during the visual imagery condition. However, an alternative interpretation is that late gamma activity could reflect cognitive aspects of the ongoing task. However, we do not have enough evidence to provide a complete explanation of this result. For instance, we do not have behavioral measures to verify that participants executed the imagery tasks with an acceptable performance. This is certainly an important limitation of this study since behavioral data and questionnaires are always important to fully understand the brain dynamics that underlie cognitive processes.

A noteworthy observation in the present results is that we did not find differences in alpha power. This frequency band has been widely associated with attentional suppression, mainly in visual modality (Toscani et al., 2010; Hanslmayr et al., 2011). Although this attentional suppression has been reported in other modalities, such as auditory and touch, these results have not been as robust as in visual modality (Foxe and Snyder, 2011). For instance, absence of differences in alpha band has been reported before for attentional tasks in auditory modality (Braboszcz and Delorme, 2011; Teng et al., 2017). Besides, alpha band has been showed to have a shared mechanism between a supra-modal and modality-specific network, which necessarily makes the auditory alpha modulation to be different from visual modality (Banerjee et al., 2011). Further research is needed to reveal the different generators and mechanisms underlying alpha modulation in the different modalities (Cohen, 2017) but this absence in results in alpha band suggests the attentional suppression mechanisms in the auditory modality operate differently from visual modality.

In the present study we observed higher theta power in passive listening compared with internally oriented attention. Theta oscillations have also been suggested to be important in auditory processing (Teng et al., 2017). Similar pattern of results were observed in a study about “inattentional deafness” where missed stimuli were indexed by reduced stimulus evoked phase synchrony in low frequencies (6–14 Hz) compared with detected stimuli, from 120 to 230 ms poststimulus onset (Callan et al., 2018). These results support that auditory stimuli might be missed during imagination processes and that frontal theta oscillation can play an important role indexing auditory attentional processing.

### Visual Imagery Exhibit a Different Pattern of Beta Oscillatory Activity

We also found early beta band differences for the visual imagery condition contrasted with inner speech and external task condition in the parietal regions (**Figure 6**).

Changes in beta band have been generally associated with top-down control deployment to maintain an internal cognitive state (Engel and Fries, 2010). However, more specifically, early beta band activity modulations have been related with an integration process between different sensory modalities (mainly auditory and visual information) during different tasks involving the input from two sensory modalities such as in McGurk illusion (audiovisual) (Roa Romero et al., 2015), perception of ambiguous audiovisual stimulus (Hipp et al., 2011) or sensory gating paradigm (auditory-somatosensory) (Kisley and Cornwell, 2006). Importantly, additional evidence for the involvement of beta band activity in multisensory processing comes from a study in which participants were instructed to respond to the appearance of auditory, visual and combined audiovisual stimuli. Only in the crossmodal condition, an enhancement was observed for beta oscillations in the time interval between 50 and 170 ms (Senkowski et al., 2006). In the case of the present study, the auditory stimuli were presented during the three conditions and there was not simultaneous visual stimulation. However, it can be assumed that during passive hearing the auditory processing was unimodal. In the case of inner speech condition, if we take into account that this kind of thought uses the auditory cortex to be performed (Shergill et al., 2001), it can also be taking as a unimodal processing of information. This is because despite the sources are different (beep/external and inner speech/internal), both are processed in the same sensory cortex. On the contrary, visual imagery has been showed to use visual cortex in order to represent visual thoughts (Kosslyn et al., 2001), and therefore, if processing of auditory stimuli is going on while visual cortex is producing visual imagery, this condition is likely to behave as crossmodal. If that were the case, then the enhanced low beta activity is indexing that visual and auditory information are being processed at the same time (Senkowski et al., 2008). There is a growing body of research providing new evidence about the importance of beta band activity in the processing of different characteristic of auditory stimuli (Cirelli et al., 2014; Alavash et al., 2017; Chang et al., 2018), and the results of the present study help to complement the findings in this line of research. Specifically, we here showed that beta increase at different range is related with the characteristics of the task, showing that during visual imagery the increase is in the full range of beta (15–30 Hz) while in the other conditions is restricted to high beta (24–30 Hz). Therefore, the different pattern of beta activity related with auditory stimuli could differentiate visual imagery from the other cognitive states.

Finally, regarding our initial hypotheses, the ERP results were different from our predictions, showing maintenance in auditory processing during imagination process. However, our predictions about gamma coincided with our results, suggesting a reduction in conscious attention during mental imagery conditions. These results suggest that ERPs and gamma oscillations are showing different aspects of auditory processing, supporting the different mechanisms underlying them, in which ERP probably is related with the brain processing while gamma activity is more associated to differences in conscious attention. Nonetheless, differences in late gamma between both mental imagery conditions were different from our predictions. Specifically, we expected a lower power in gamma during inner speech than during visual imagery based on the theoretical framework that if the modality of thought is the same that incoming stimulation, there should be a competition for processing resources (Villena-González et al., 2016). However, the inversed effect was observed, which strongly suggest that attentional suppression mechanisms operate different in auditory modality compared to visual modality. Therefore, this results provide evidence against the view that attentional mechanisms are supra-modal (Farah et al., 1989; Eimer and Van Velzen, 2002) and also clarify that the “modality resource competition” between visual imagery and visual perception described in Villena-González et al. (2016) do not occur for the auditory modality.

## Conclusion

In summary, our event-related potential results showed that when attention is inwardly oriented to visual or verbal/auditory imagery, the processing of auditory stimuli is maintained compared with a passive listening condition. Our results in the frequency domain showed early changes in frontal gamma and theta power which reflects differences between external and internal attention. Specifically, the reduced amplitude in gamma and theta band during inward attention may reflect reduced conscious attention of the current stimulation. Furthermore, different pattern of beta activity was observed during visual imagery which differentiate this condition from the other ones and it could reflect crossmodal integration between visual and auditory modalities. Finally, our work provides more evidence to confirm the differential electrophysiological dynamics underlying the visual and auditory/verbal imagery.

## Author Contributions

MV-G, IP-G, VL, and ER conceived and designed the experiments. MV-G and IP-G performed the experiments and analyzed the data. MV-G, VL, and ER contributed reagents, materials, and analysis tools. MV-G wrote the paper.

## Conflict of Interest Statement

The authors declare that the research was conducted in the absence of any commercial or financial relationships that could be construed as a potential conflict of interest.

## References

[B1] Aboitiz F. (2012). Gestures, vocalizations, and memory in language origins. *Front. Evol. Neurosci.* 4:2. 10.3389/fnevo.2012.00002 22347184 PMC3269654

[B2] Alavash M. Daube C. Wostmann M. Brandmeyer A. Obleser J. (2017). Large-scale network dynamics of beta-band oscillations underlie auditory perceptual decision-making. *Netw. Neurosci.* 1 166–191. 10.1162/NETN_a_00009 29911668 PMC5988391

[B3] Baird B. Smallwood J. Lutz A. Schooler J. W. (2014). The decoupled mind: mind-wandering disrupts cortical phase-locking to perceptual events. *J. Cogn. Neurosci.* 26 2596–2607. 10.1162/jocn_a_00656 24742189

[B4] Banerjee S. Snyder A. C. Molholm S. Foxe J. J. (2011). Oscillatory alpha-band mechanisms and the deployment of spatial attention to anticipated auditory and visual target locations: supramodal or sensory-specific control mechanisms? *J. Neurosci.* 31 9923–9932. 10.1523/JNEUROSCI.4660-10.2011 21734284 PMC3343376

[B5] Barron E. Riby L. M. Greer J. Smallwood J. (2011). Absorbed in thought: the effect of mind wandering on the processing of relevant and irrelevant events. *Psychol. Sci.* 22 596–601. 10.1177/0956797611404083 21460338

[B6] Bekinschtein T. A. Dehaene S. Rohaut B. Tadel F. Cohen L. Naccache L. (2009). Neural signature of the conscious processing of auditory regularities. *Proc. Natl. Acad. Sci. U.S.A.* 106 1672–1677. 10.1073/pnas.0809667106 19164526 PMC2635770

[B7] Bosman C. A. Schoffelen J. M. Brunet N. Oostenveld R. Bastos A. M. Womelsdorf T. (2012). Attentional stimulus selection through selective synchronization between monkey visual areas. *Neuron* 75 875–888. 10.1016/j.neuron.2012.06.037 22958827 PMC3457649

[B8] Braboszcz C. Delorme A. (2011). Lost in thoughts: neural markers of low alertness during mind wandering. *Neuroimage* 54 3040–3047. 10.1016/j.neuroimage.2010.10.008 20946963

[B9] Callan D. E. Gateau T. Durantin G. Gonthier N. Dehais F. (2018). Disruption in neural phase synchrony is related to identification of inattentional deafness in real-world setting. *Hum. Brain Mapp.* 39 2596–2608. 10.1002/hbm.24026 29484760 PMC6866488

[B10] Castelhano J. Rebola J. Leitao B. Rodriguez E. Castelo-Branco M. (2013). To perceive or not perceive: the role of gamma-band activity in signaling object percepts. *PLoS One* 8:e66363. 10.1371/journal.pone.0066363 23785494 PMC3681966

[B11] Cervenka M. C. Nagle S. Boatman-Reich D. (2011). Cortical high-gamma responses in auditory processing. *Am. J. Audiol.* 20 171–180. 10.1044/1059-0889(2011/10-0036) 22158634 PMC3848128

[B12] Chang A. Bosnyak D. J. Trainor L. J. (2018). Beta oscillatory power modulation reflects the predictability of pitch change. *Cortex* 106 248–260. 10.1016/j.cortex.2018.06.008 30053731

[B13] Cirelli L. K. Bosnyak D. Manning F. C. Spinelli C. Marie C. Fujioka T. (2014). Beat-induced fluctuations in auditory cortical beta-band activity: using EEG to measure age-related changes. *Front. Psychol.* 5:742. 10.3389/fpsyg.2014.00742 25071691 PMC4093753

[B14] Cohen M. X. (2014a). *Chapter 2: Advantages and Limitations of Time- and Time-Frequency Domain Analyses Analyzing Neural Time Series Data : Theory and Practice.* Cambridge, MA: MIT Press, 21–24.

[B15] Cohen M. X. (2014b). *Chapter 33: Nonparametric Permutation Testing Analyzing Neural Time Series Data : Theory and Practice.* Cambridge, MA: MIT Press, 459–471.

[B16] Cohen M. X. (2017). Where does EEG come from and what does it mean? *Trends Neurosci.* 40 208–218. 10.1016/j.tins.2017.02.004 28314445

[B17] Cooper N. R. Croft R. J. Dominey S. J. Burgess A. P. Gruzelier J. H. (2003). Paradox lost? Exploring the role of alpha oscillations during externally vs. internally directed attention and the implications for idling and inhibition hypotheses. *Int. J. Psychophysiol.* 47 65–74. 12543447 10.1016/s0167-8760(02)00107-1

[B18] Debener S. Herrmann C. S. Kranczioch C. Gembris D. Engel A. K. (2003). Top-down attentional processing enhances auditory evoked gamma band activity. *Neuroreport* 14 683–686. 10.1097/01.wnr.0000064987.96259.5c 12692463

[B19] Delorme A. Makeig S. (2004). EEGLAB: an open source toolbox for analysis of single-trial EEG dynamics including independent component analysis. *J. Neurosci. Methods* 134 9–21. 10.1016/j.jneumeth.2003.10.009 15102499

[B20] Eimer M. Van Velzen J. (2002). Crossmodal links in spatial attention are mediated by supramodal control processes: evidence from event-related potentials. *Psychophysiology* 39 437–449. 12212636 10.1017/S0048577201393162

[B21] Engel A. K. Fries P. (2010). Beta-band oscillations–signalling the status quo? *Curr. Opin. Neurobiol.* 20 156–165. 10.1016/j.conb.2010.02.015 20359884

[B22] Farah M. J. Wong A. B. Monheit M. A. Morrow L. A. (1989). Parietal lobe mechanisms of spatial attention: modality-specific or supramodal? *Neuropsychologia* 27 461–470. 2733819 10.1016/0028-3932(89)90051-1

[B23] Foxe J. J. Snyder A. C. (2011). The role of alpha-band brain oscillations as a sensory suppression mechanism during selective attention. *Front. Psychol.* 2:154. 10.3389/fpsyg.2011.00154 21779269 PMC3132683

[B24] Golchert J. Smallwood J. Jefferies E. Seli P. Huntenburg J. M. Liem F. (2017). Individual variation in intentionality in the mind-wandering state is reflected in the integration of the default-mode, fronto-parietal, and limbic networks. *Neuroimage* 146 226–235. 10.1016/j.neuroimage.2016.11.025 27864082

[B25] Hanslmayr S. Gross J. Klimesch W. Shapiro K. L. (2011). The role of alpha oscillations in temporal attention. *Brain Res. Rev.* 67 331–343. 10.1016/j.brainresrev.2011.04.002 21592583

[B26] Hipp J. F. Engel A. K. Siegel M. (2011). Oscillatory synchronization in large-scale cortical networks predicts perception. *Neuron* 69 387–396. 10.1016/j.neuron.2010.12.027 21262474

[B27] Kam J. W. Dao E. Farley J. Fitzpatrick K. Smallwood J. Schooler J. W. (2011). Slow fluctuations in attentional control of sensory cortex. *J. Cogn. Neurosci.* 23 460–470. 10.1162/jocn.2010.21443 20146593

[B28] Kam J. W. Dao E. Stanciulescu M. Tildesley H. Handy T. C. (2013). Mind wandering and the adaptive control of attentional resources. *J. Cogn. Neurosci.* 25 952–960. 10.1162/jocn_a_00375 23448525

[B29] Kam J. W. Handy T. C. (2013). The neurocognitive consequences of the wandering mind: a mechanistic account of sensory-motor decoupling. *Front. Psychol.* 4:725. 10.3389/fpsyg.2013.00725 24133472 PMC3796327

[B30] Keil A. Debener S. Gratton G. Junghofer M. Kappenman E. S. Luck S. J. (2014). Committee report: publication guidelines and recommendations for studies using electroencephalography and magnetoencephalography. *Psychophysiology* 51 1–21. 10.1111/psyp.12147 24147581

[B31] Kisley M. A. Cornwell Z. M. (2006). Gamma and beta neural activity evoked during a sensory gating paradigm: effects of auditory, somatosensory and cross-modal stimulation. *Clin. Neurophysiol.* 117 2549–2563. 10.1016/j.clinph.2006.08.003 17008125 PMC1773003

[B32] Kosslyn S. M. Ganis G. Thompson W. L. (2001). Neural foundations of imagery. *Nat. Rev. Neurosci.* 2 635–642. 10.1038/35090055 11533731

[B33] Luck S. J. (2005). *An Introduction to the Event-Related Potential Technique.* Cambridge, MA: MIT Press.

[B34] Maris E. Oostenveld R. (2007). Nonparametric statistical testing of EEG- and MEG-data. *J. Neurosci. Methods* 164 177–190. 10.1016/j.jneumeth.2007.03.024 17517438

[B35] Nichols T. E. Holmes A. P. (2002). Nonparametric permutation tests for functional neuroimaging: a primer with examples. *Hum. Brain Mapp.* 15 1–25. 11747097 10.1002/hbm.1058 PMC6871862

[B36] Oostenveld R. Fries P. Maris E. Schoffelen J. M. (2011). FieldTrip: open source software for advanced analysis of MEG, EEG, and invasive electrophysiological data. *Comput. Intell. Neurosci.* 2011:156869. 10.1155/2011/156869 21253357 PMC3021840

[B37] Peirce J. W. (2007). PsychoPy–psychophysics software in python. *J. Neurosci. Methods* 162 8–13. 10.1016/j.jneumeth.2006.11.017 17254636 PMC2018741

[B38] Roa Romero Y. Senkowski D. Keil J. (2015). Early and late beta-band power reflect audiovisual perception in the McGurk illusion. *J. Neurophysiol.* 113 2342–2350. 10.1152/jn.00783.2014 25568160 PMC4416591

[B39] Rodriguez E. George N. Lachaux J. P. Martinerie J. Renault B. Varela F. J. (1999). Perception’s shadow: long-distance synchronization of human brain activity. *Nature* 397 430–433. 10.1038/17120 9989408

[B40] Seli P. Risko E. F. Smilek D. (2016a). On the necessity of distinguishing between unintentional and intentional mind wandering. *Psychol. Sci.* 27 685–691. 10.1177/0956797616634068 26993740

[B41] Seli P. Risko E. F. Smilek D. Schacter D. L. (2016b). Mind-wandering with and without intention. *Trends Cogn. Sci.* 20 605–617. 10.1016/j.tics.2016.05.010 27318437 PMC5004739

[B42] Senkowski D. Molholm S. Gomez-Ramirez M. Foxe J. J. (2006). Oscillatory beta activity predicts response speed during a multisensory audiovisual reaction time task: a high-density electrical mapping study. *Cereb. Cortex* 16 1556–1565. 10.1093/cercor/bhj091 16357336

[B43] Senkowski D. Schneider T. R. Foxe J. J. Engel A. K. (2008). Crossmodal binding through neural coherence: implications for multisensory processing. *Trends Neurosci.* 31 401–409. 10.1016/j.tins.2008.05.002 18602171

[B44] Shergill S. S. Bullmore E. T. Brammer M. J. Williams S. C. Murray R. M. McGuire P. K. (2001). A functional study of auditory verbal imagery. *Psychol. Med.* 31 241–253. 11232912 10.1017/s003329170100335x

[B45] Smallwood J. Beach E. Schooler J. W. Handy T. C. (2008). Going AWOL in the brain: mind wandering reduces cortical analysis of external events. *J. Cogn. Neurosci.* 20 458–469. 10.1162/jocn.2008.20037 18004943

[B46] Smallwood J. Schooler J. W. (2006). The restless mind. *Psychol. Bull.* 132 946–958. 10.1037/0033-2909.132.6.946 17073528

[B47] Smallwood J. Schooler J. W. (2015). The science of mind wandering: empirically navigating the stream of consciousness. *Annu. Rev. Psychol.* 66 487–518. 10.1146/annurev-psych-010814-015331 25293689

[B48] Teng X. Tian X. Rowland J. Poeppel D. (2017). Concurrent temporal channels for auditory processing: oscillatory neural entrainment reveals segregation of function at different scales. *PLoS Biol.* 15:e2000812. 10.1371/journal.pbio.2000812 29095816 PMC5667736

[B49] Tiitinen H. Sinkkonen J. Reinikainen K. Alho K. Lavikainen J. Naatanen R. (1993). Selective attention enhances the auditory 40-Hz transient response in humans. *Nature* 364 59–60. 10.1038/364059a0 8316297

[B50] Toscani M. Marzi T. Righi S. Viggiano M. P. Baldassi S. (2010). Alpha waves: a neural signature of visual suppression. *Exp. Brain Res.* 207 213–219. 10.1007/s00221-010-2444-7 20972777

[B51] Villena-González M. López V. Rodríguez E. (2016). Orienting attention to visual or verbal/auditory imagery differentially impairs the processing of visual stimuli. *Neuroimage* 132 71–78. 10.1016/j.neuroimage.2016.02.013 26876471

