# Supplementary material for: Beta Oscillations Distinguish Between Two Forms of Mental Imagery While Gamma and Theta Activity Reflects Auditory Attention
Source: Front Hum Neurosci. 2018 Sep 25;12:389. doi: 10.3389/fnhum.2018.00389 (PMC6178143; doi:10.3389/fnhum.2018.00389)
Supplement: Supplementary file 1 [file Data_Sheet_1.docx]

This is the supplementary material for the article entitled “Beta oscillations distinguish between two forms of mental imagery while gamma and theta activity reflects auditory attention” Villena-Gonzalez, et al. under review in the Journal Frontiers in Human Neuroscience.

**P200 analysis**

Mean amplitude of P2 (between 200-250ms) evoked by auditory probes were also compared with repeated-measures ANOVA using Electrode (three levels: Fz, FCz, Cz) and Condition (three levels: visual imagery, inner speech, passive listening) as factors. The ANOVA analysis was carried out using STATISTICA 7.0 software (StatSoft, Inc). The result of this analysis showed no significant differences P1 (F(4,49)= 1,23, p=0,303).

**Supplementary figures**


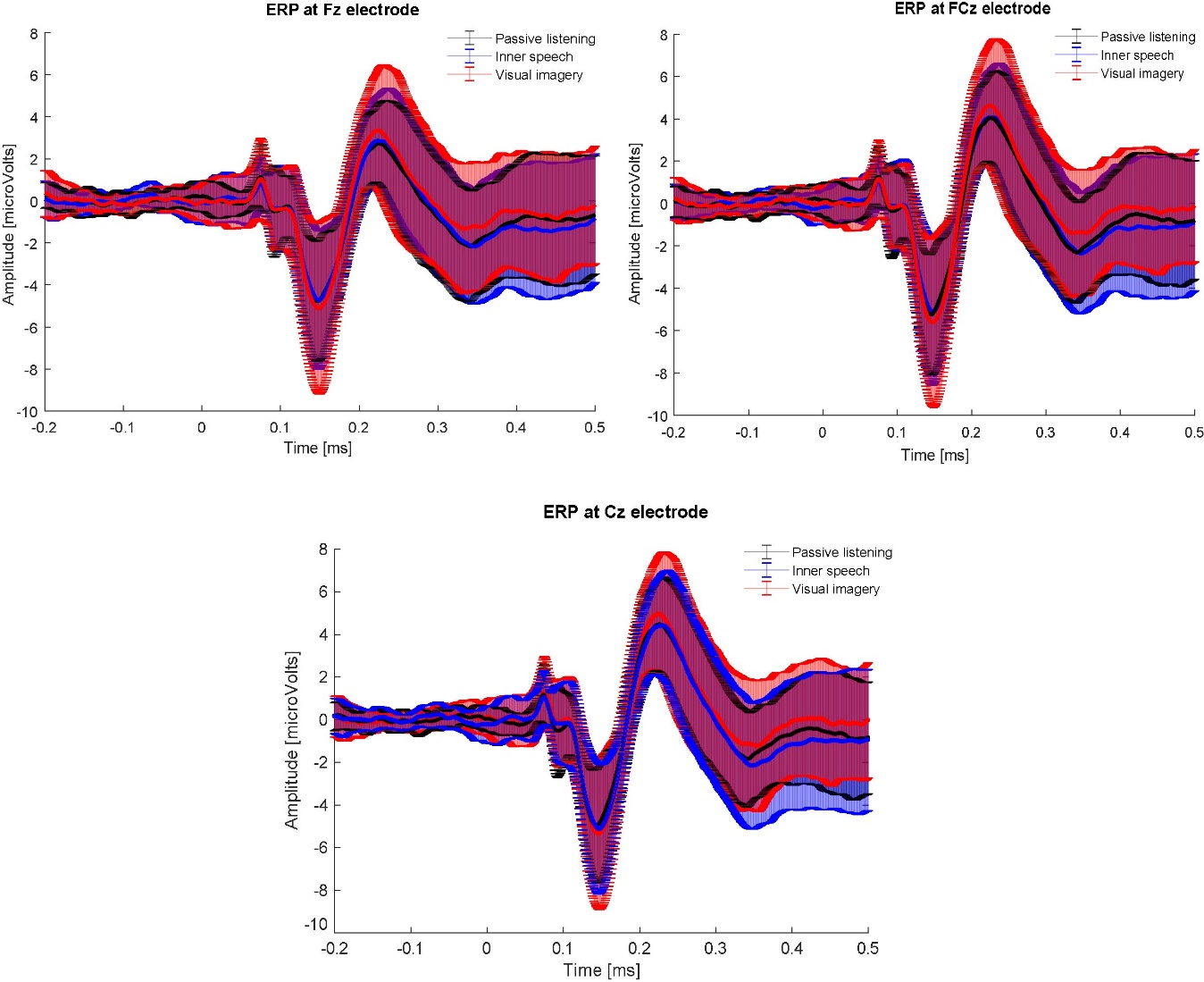


**Figure S1.** **ERP waveform with standard deviation**. ERP waveforms for three conditions are shown for different midline fronto-central electrodes; Fz, FCz and Cz. Standard deviation are also depicted. There is no difference between conditions for any of the early sensory components of ERP.


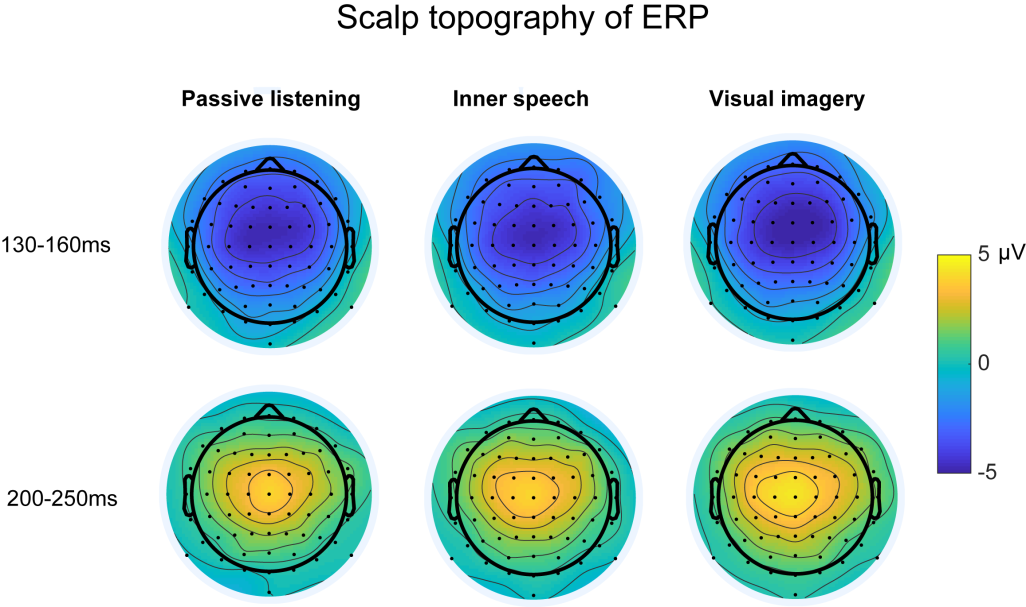


**Figure S2. Scalp topography of early components of ERP.** Scalp topography is shown for the three conditions during the time window of N1 (130-160ms) and P2 (200-250ms)


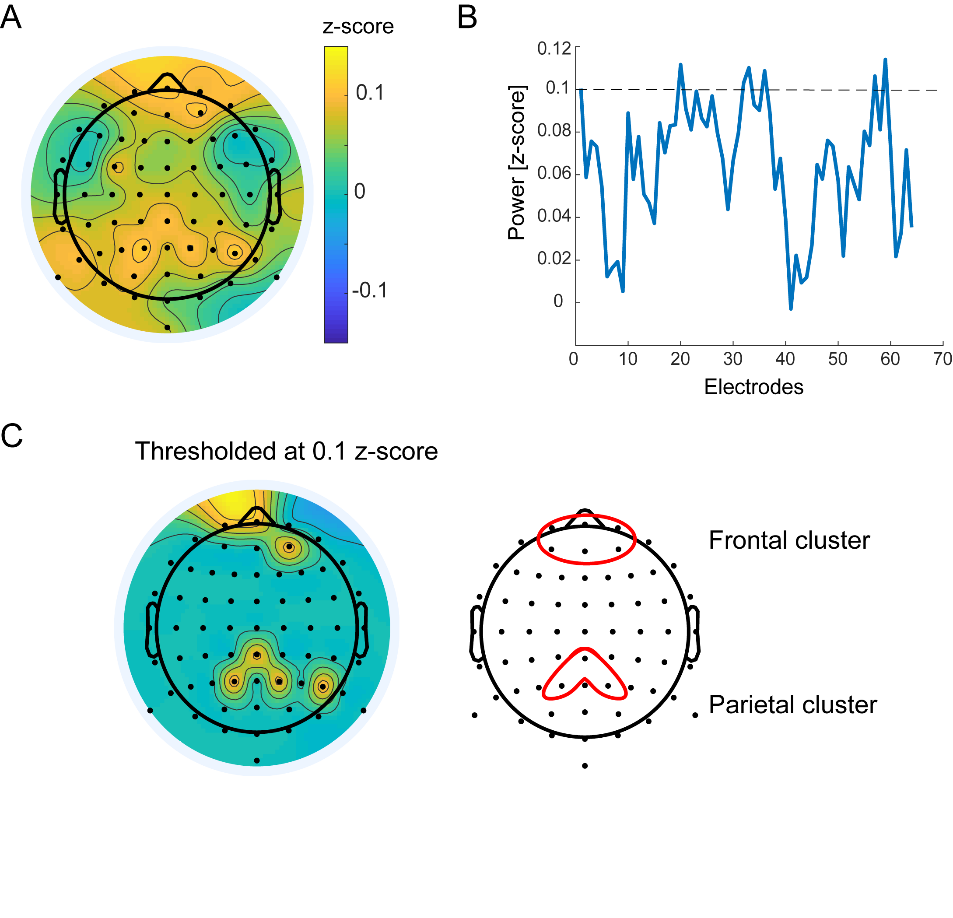


**Figure S3. Cluster selection based on scalp distribution of power.** A) Scalp distribution of spectral power after averaging across epochs, time, frequency, subjects and conditions. B) Mean of power for all the electrodes is shown. The threshold (0.1 z-score) was selected in order to keep the 10 percent of the electrodes showing the maximum values of spectral power. C) Scalp topography after applying the threshold (left). Values under the threshold were converted to zero. Two clusters can be observed; frontal and parietal (right).


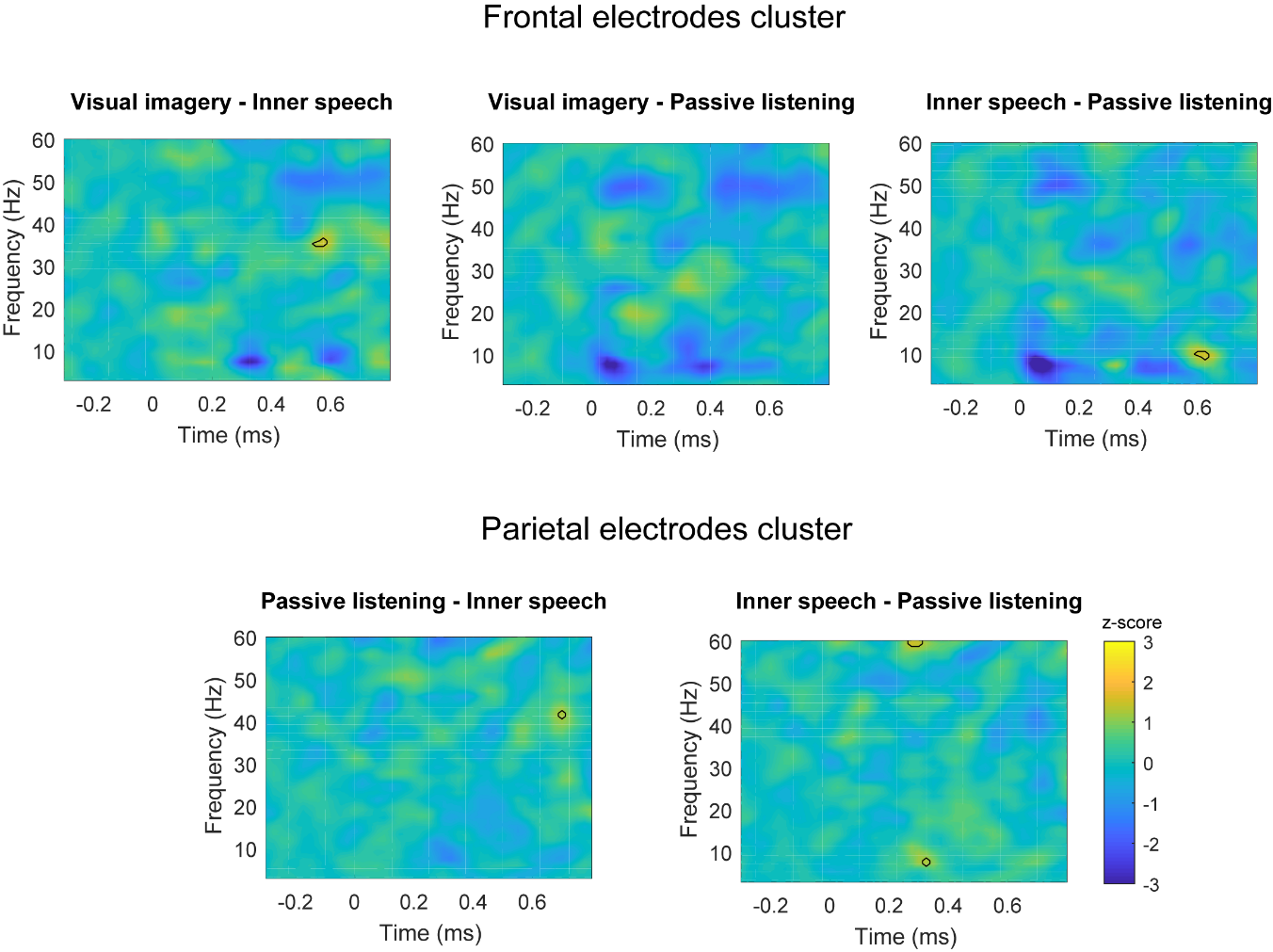


**Figure S4. Time-frequency plots showing no difference in frontal and parietal regions.** Upper panel: Time-frequency power subtraction between visual imagery and inner speech (left), visual imagery and passive listening (middle) and inner speech and passive listening (right). Lower panel: Time-frequency power subtraction between passive listening and inner speech (left) and inner speech and passive listening (right). Nonparametric permutation test and multiple comparison corrections was performed.

**
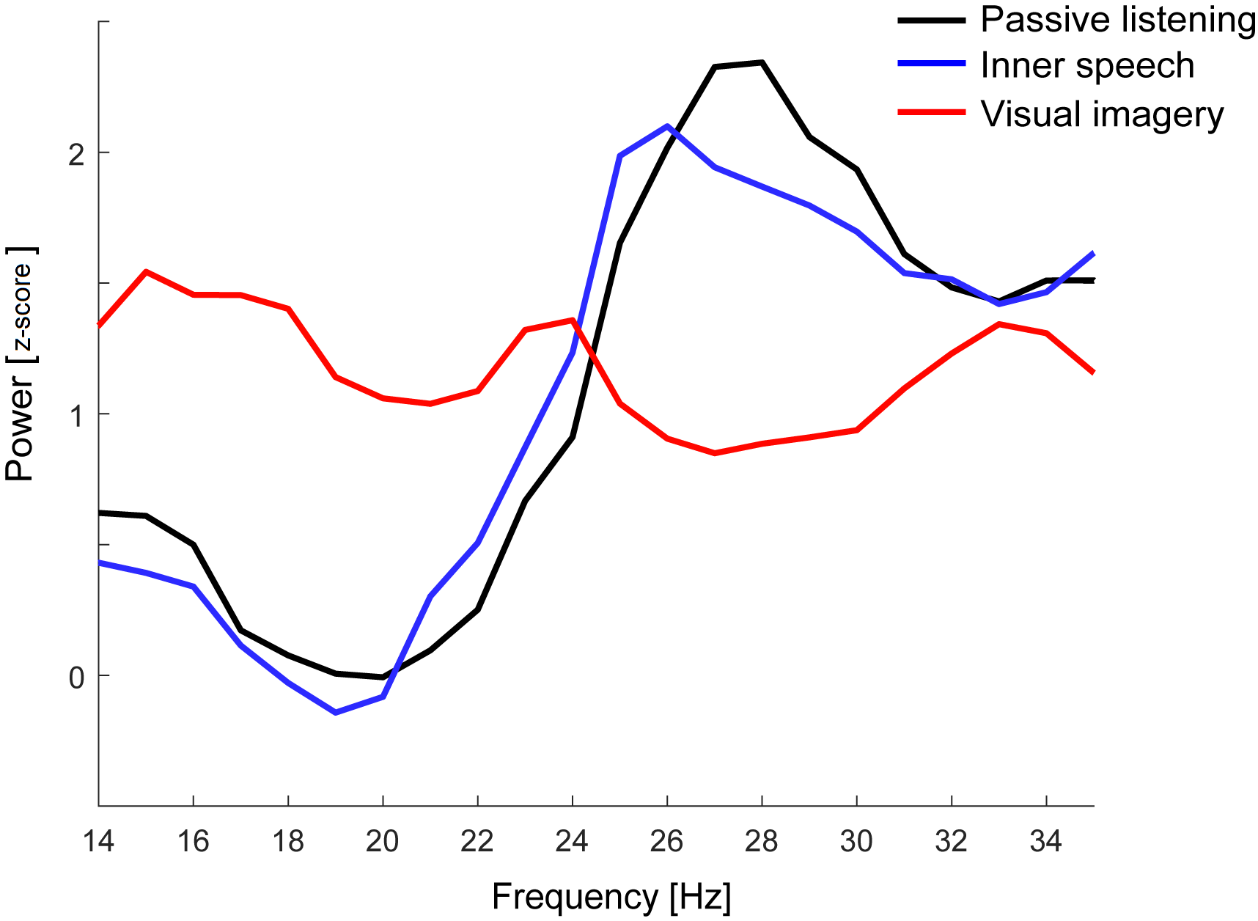
**

**Figure S5. Spectral power of beta band for each experimental condition.**
